# Supplementary material for: Microglia-like Cells Promote Neuronal Functions in Cerebral Organoids
Source: Cells. 2021 Dec 30;11(1):124. doi: 10.3390/cells11010124 (PMC8750120; doi:10.3390/cells11010124)
Supplement: Supplementary file 1 [file cells-11-00124-s001.zip › cells-1513262-supplementary.pdf]

**Supplementary Materials:**

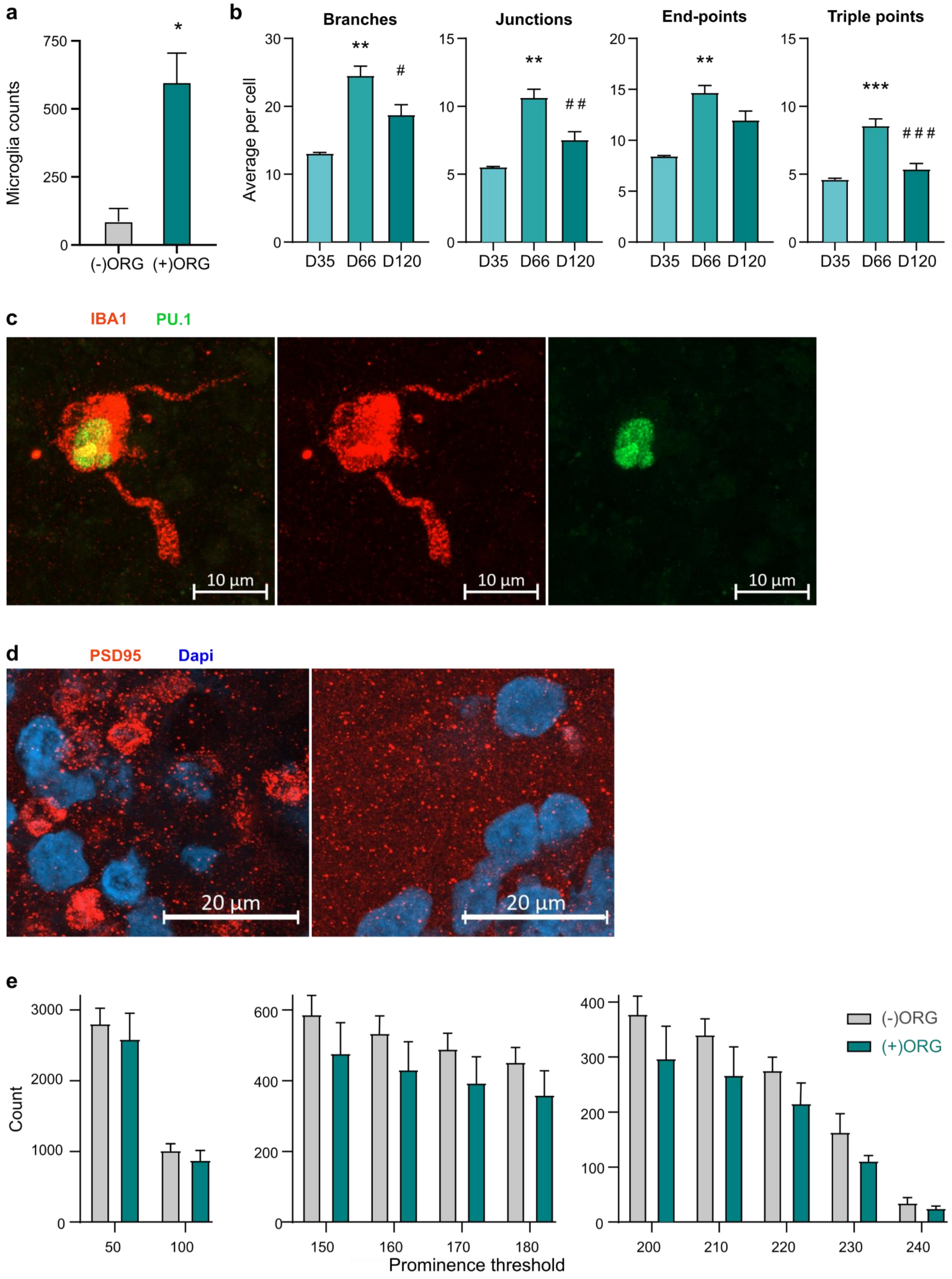

**Figure S1.** (**a**) The sections from (+)ORGs hold significantly more IBA+ cells than from (-)ORGs. The data bars present means (+/- standard error of mean) from five (-)ORGs against 18 (+)ORGs. (**b**) Skeletal analysis of IBA1+ cells in organoids at 35 DIV, 66 DIV and 120 DIV, showed a significant increase in complexity from day 35 to 66 but decreased from day 66 to 120. The bars represent means (+/- SEM) from three organoids at 35 DIV, six at 66 DIV and nine at 120 DIV. Significance ***p < 0.001, **p < 0.01, *p < 0.05 compared to D35, # indicate similarly significance compared to D66 (Tukey test, p>0.05, ns). The bars represent the mean (+/- SEM) percentage of three organoids for the day 35 group, six for day 66 and nine for day 120. (**c**) Representative orthogonal projection image of a single cell double-positive for IBA1 and PU.1 found in an immunostained slice of an organoid at 120 DIV. (**d**) Immuno-histochemical stainings of day 120 cerebral organoid cryosections show PSD95 localization as scattered puncta in post-synaptic termini, and in cell soma. (**e**) Counts of PSD95+ puncta in orthogonal projection images of PSD95 immunohistochemical stainings, presented for incrementally increasing thresholds of puncta prominence. The bars present means (+/- SEM) of 16 identically acquired confocal orthogonal projection images of immunohistochemical stainings against PSD95 in four organoids at 120 DIV. The X-axis shows the applied prominence threshold, while the Y-axis presents the number of puncta the algorithm detected in a particular image at a particular prominence threshold. Mann-Whitney unpaired test, p>0.05, ns. The results in panels a and b come from organoids of the iPCS line MBE2968, while c-e come from MAD6.

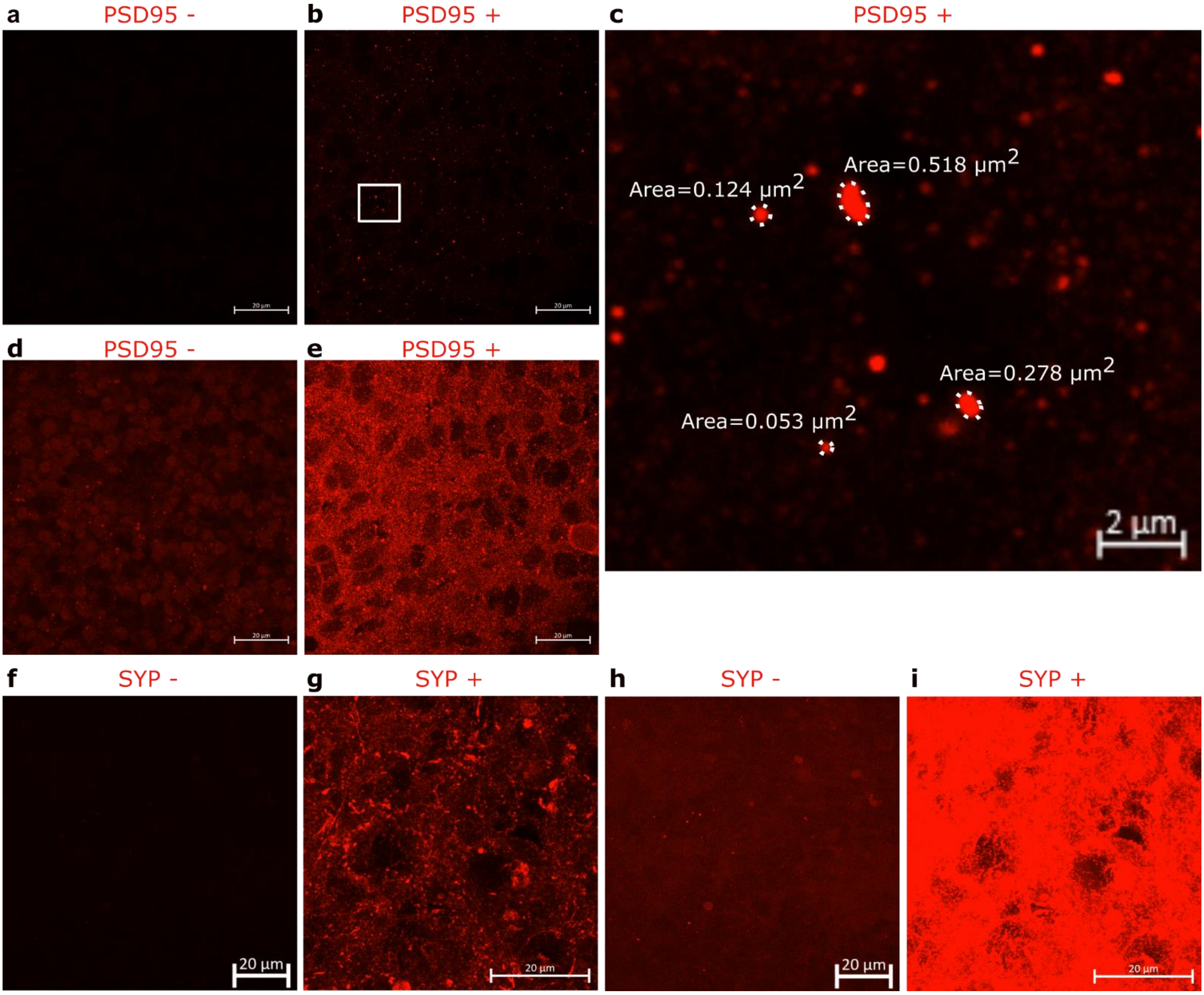


**Figure S2.** (**a**) Negative control for the IHC detection of PSD95, no primary antibody, brightness unmodified. (**b**) IHC detection of PSD95, brightness unmodified. (**c**) Close-up to the rectangle drawn on figure b, with areas of representative puncta measured (using Zen 3.2 blue edition). (**d**) The same image as in figure a, with increased brightness to detect the background fluorescence. (**e**) The same image as in figure b, with brightness increase identical to figure d. (**f**) Negative control for the IHC detection of SYP, no primary antibody, brightness increased to the same level as show in the IBA1 and SYP IHC in Figure 2b. (**g**) IHC detection of SYP, brightness increased to the same level as in figure f. (**h**) The same image as in figure f, with increased brightness to detect the background fluorescence. (**i**) The same image as in figure g with brightness increase identical to figure h. All brightness modifications were performed in the Zen 3.2 blue edition by adjusting the white levels. All PSD95 and SYP images were acquired using identical settings on the confocal microscope to allow comparison between the figures. All images come from organoids of the iPCS line MBE2968.

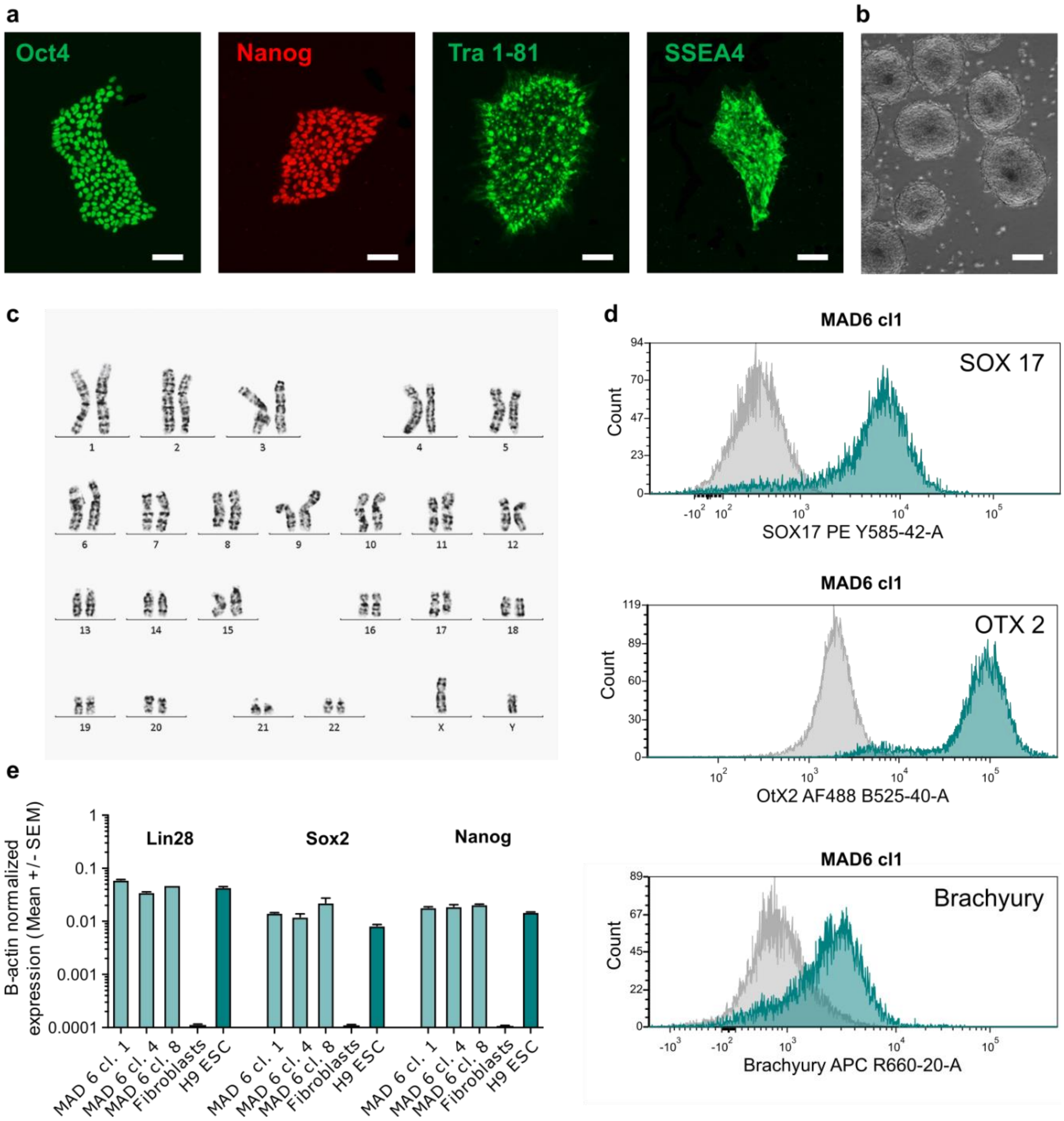


**Figure S3.** (**a**) IHC detection of the pluripotency markers of Oct4, Nanog, Tra 1-81 and SSEA4 expressed in iPCS colonies. Scale bars 100µm. (**b**) Generation of embryoid bodies to test for differentiation to all three embryonic layers. Scale bar 50µm. (**c**) Karyotyping results showed normal 46 XY karyotype. (**d**) Detection of markers expressed in endodermal (SOX17), ectodermal (OTX2) and mesodermal (BRACHYURY) lineages, measured in flowcytometry analysis of single cell suspension of the embryoid bodies. (**e**) RT-qPCR measurements of the expression of the pluripotency markers *Lin28*, *Sox2* and *Nanog* across three clones of MAD6, with fibroblasts and an embryonic stem cell line (H9 ESC) as negative and positive controls, respectively.
